# Performance tracking in female youth soccer through wearables and subjective assessments

**DOI:** 10.3389/fspor.2025.1627820

**Published:** 2025-06-30

**Authors:** Stefan Kranzinger, Christina Kranzinger, Wolfgang Kremser, Burkhard Duemler

**Affiliations:** ^1^ Human Motion Analytics, Salzburg Research Forschungsgesellschaft mbH, Salzburg, Austria; ^2^ Athlete Performance, Adidas AG, Herzogenaurach, Germany

**Keywords:** youth soccer performance, wearable sensors, subjective training assessment, machine learning in sports, longitudinal monitoring

## Abstract

This study investigates performance development and the relationship between subjective and objective training assessments in female youth soccer using wearable sensor technology. The aim of this study was to assess how subjective post-training ratings (intensity and happiness) relate to high-percentile performance outputs, and to identify longitudinal trends in female youth soccer players using IMU-based wearable data. Data were collected over a 14-month period from 46 players (U17 and U20 teams) equipped with foot-mounted inertial measurement units (IMUs) during regular training sessions. Objective performance metrics, including 95th percentile of ball speed, peak speed, and absolute distance, were derived using a multi-stage machine learning pipeline, while subjective metrics (intensity and happiness) were collected via post-session Likert-scale questionnaires using an app. Using the modified Mann-Kendall test, we found 30 significant longitudinal trends, with 14 positive and 16 negative trends across key performance metrics. Peak speed showed the highest number of trends (13), followed by absolute distance (10) and ball speed (7). Correlation analyses based on the Spearman coefficient (with False Discovery Rate correction) revealed meaningful associations between subjective self-assessments and high-percentile performance metrics, with notable differences across player positions and age groups. A robustness check confirmed these patterns also hold when analyzing the 99th percentile of performance outputs. Our findings underscore the value of combining wearable sensor data with subjective evaluations for individualized, role-specific performance monitoring and training optimization in youth soccer. However, as an exploratory study with a single cohort, findings require further validation in broader populations.

## 1 Introduction

### 1.1 General context

Wearable sensors, or *wearables*, can be unobtrusively worn on the body and are increasingly used in soccer training to collect data on players’ physiological and physical activity (1). For example, (2) presents a wearable GNSS platform that calculates a player’s total distance covered and the speed range in which they covered it (walking, jogging, fast running, sprinting), along with their top speed and other sprint statistics. Similar metrics can be calculated using the player’s inertial data (three-dimensional acceleration and angular velocity), measured by an inertial measurement unit (IMU). A wrist-worn inertial data-based system is presented in (3), where in addition to the aforementioned speed metrics, the authors estimate the player-ball interaction (pass, kick, dribble). Furthermore, they differentiate their analysis based on player position (defender, midfielder, striker). This added information can be used by athletes and their coaches for *training load monitoring*.

### 1.2 Specific problem and gaps in the literature

Training load (TL) has an internal and an external component (4). The internal component includes all physiological and psychological stressors that act upon the athlete. The external component is the work performed during training/competition. TL monitoring aims to quantify the relationship between TL and performance, as well as the relationship between TL and injury risk (4, 5). While it is believed necessary to overload athletes to some degree during training to improve their performance capacity (6), overdoing it increases injury risk (4) and, in extreme cases, leads to a permanent decrease of performance capacity due to overtraining (7).

While wearables are capable of directly or indirectly measuring physiological stressors (through e.g., a wearable heart rate monitor) and external TL measures like total distance and speed parameters, they are limited when it comes to measuring psychological stressors, i.e., subjective TL. The most common measure of subjective training load is a *sessional rating of perceived exertion* (sPRE) (5). It is important that an sPRE follows a non-linear scale with verbal anchors (5). Such a scale is the CR10 scale (8) where expressions such as “extremely weak,” “weak,” “moderate” and “very strong” map to a zero-to-ten number range in a non-equidistant manner. To provide a more fine-grained picture of training load, questionnaires may use a 0–100 range (CR100) and split the sRPE into separate scores for e.g., breathlessness, upper body exertion, and cognitive exertion (9). While most subjective training load measures focus on perceived exertion, emotional responses to performance, such as satisfaction or happiness, can also influence motivation, recovery behavior, and long-term engagement in sport. Positive affective states have been shown to modulate perceived readiness and performance, especially in young or developing athletes. A recent systematic review highlights that psychological well-being and pleasant emotions not only complement negative affect but can serve as performance-related motivational drivers in sport (10). Furthermore, empirical evidence from a large-scale study among collegiate athletes indicates that subjective happiness is significantly associated with concentration regulation and need satisfaction, both of which affect performance and are psychologically relevant (11). Therefore, we included a post-session “happiness” rating to capture players’ emotional responses to training, which may reflect self-perceived performance satisfaction, confidence, and overall psychological readiness.

Understanding the interplay between subjective perceptions of TL and objective performance metrics is crucial for the further development of athletes and the optimization of training strategies in elite sport (12). In their study of TL monitoring for youth soccer players, (13) conclude that both external and internal TL measures should be considered by trainers when designing training programs. Our study addresses this need by using data collected from a wearable insole with integrated IMU to analyze performance metrics in female youth soccer players and questionnaires.

### 1.3 Study aim

The aim of this study is twofold: (1) to identify longitudinal trends in performance development among female youth soccer players, and (2) to investigate the relationship between subjective self-assessments (intensity and happiness) and objective high-percentile performance metrics derived from wearable IMU data.

This approach provides valuable insights into the development of athletes and contributes to more effective and individualized performance monitoring in sports science.

## 2 Materials and methods

### 2.1 Data

#### 2.1.1 Data collection

Data were collected between November 7, 2023 and January 10, 2025 from 46 female youth soccer players (U17 and U20) (age: avg 16.0, sd = 1.4). Note that two players trained in both teams, and thus they appear twice in inter-team comparisons. This study was conducted in Germany and used secondary, anonymized data collected independently of this study as part of an ongoing project by Adidas AG. Participation in the data collection was voluntary and conducted in compliance with German and EU legal requirements. Prior to data collection, participants (and, for minors, their legal guardians) provided informed consent, including agreement to a privacy notice which explicitly allows the use of anonymized data for scientific research. To evaluate whether a formal ethics vote was required, we consulted the ethical guidelines published by the German Council for Social and Economic Data (RatSWD; https://doi.org/10.17620/02671.1) and concluded that none was required. Data processing was conducted in accordance with the EU General Data Protection Regulation (GDPR, Regulation (EU) 2016/679).

**Figure 1:**
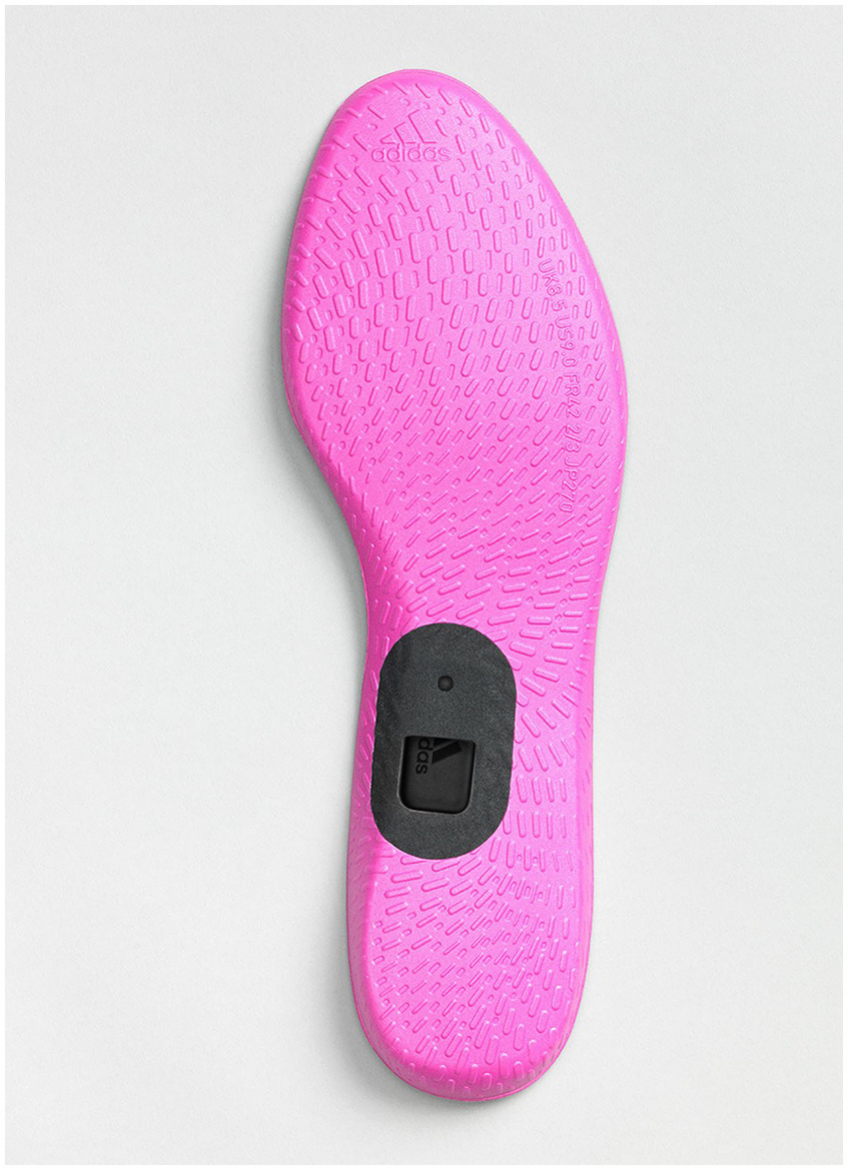
Team FX sensor insole.

**Figure 2:**
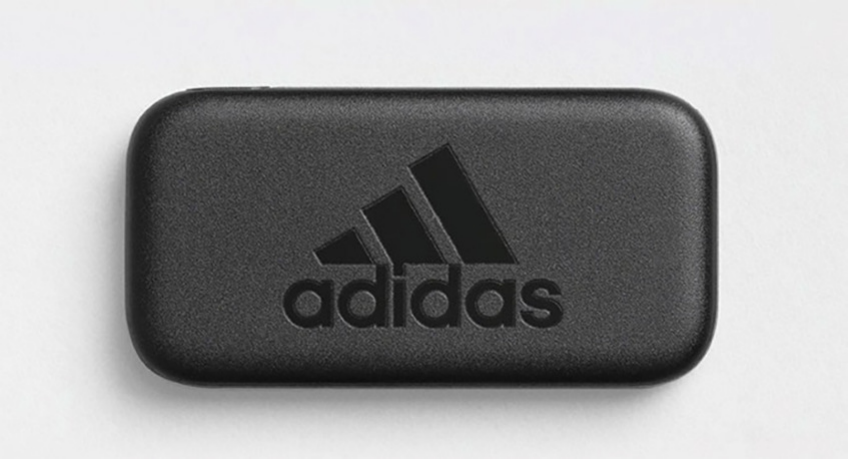
IMU, Jacquard Tag (Google LLC, USA).

During a regular training session, the 3D acceleration and 3D angular velocity of the dominant foot were recorded at 200 Hz with an *adidas TEAM-FX* insole (Figure 1). The insole housed a *Jacquard Tag* IMU (Figure 2) in a small cavity under the arch of the dominant foot. This sensor position is recommended by (14) and guarantees that the sensor does not poke into the foot. The size of the sensor unit was 38.8 × 19.7 × 5.8 mm with a weight of 5.5 g. The recorded IMU data were buffered on the Jacquard Tag for further processing.

#### 2.1.2 Sensor data processing and calculation of performance metrics

**Figure 3:**
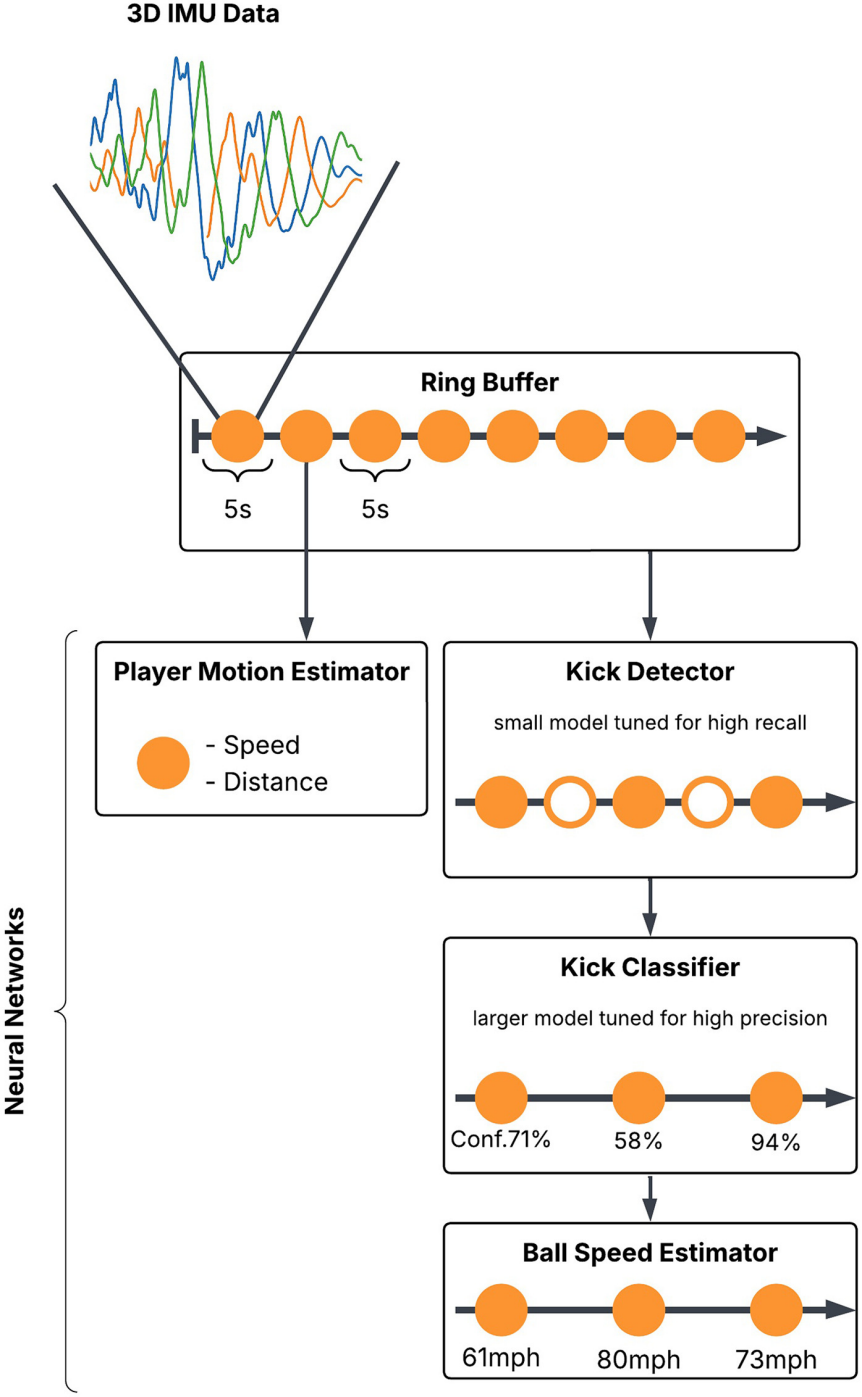
The machine learning pipeline for processing the IMU data.

The data was processed with the Jacquard Tag’s inbuilt processor to calculate football-relevant metrics using implemented machine learning algorithms partially based on the work of (15, 16). To derive the objective performance metrics **absolute distance, ball speed, and peak speed**, raw sensor data from IMUs was processed using a multi-stage classification and segmentation approach illustrated in Figure 3. The system integrates machine learning algorithms to detect and classify ball interactions and player movement. The machine learning models used to derive performance metrics (e.g., ball speed, peak speed) were provided as part of the TEAM FX insole platform and were not developed or modified by the authors. Consequently, we did not have access to internal model architectures or training procedures, and no additional model validation or tuning was performed in the context of this study.

The data set for *absolute distance* and *peak speed* was structured to capture key performance metrics at 5 s intervals. The absolute distance covered by a player during each interval can therefore be derived directly from the average speed. In addition, whenever a ball was passed or kicked during the training, the ball speed at that moment of passing or kicking was recorded and stored separately in the dataset. This allowed precise analysis of ball-related actions in conjunction with player movement data. This data structure provides a comprehensive framework for analysing both continuous movement patterns and discrete ball interactions during the training. This system follows a structured pipeline consisting of:

• **Kick detection and classification:** A *Kick Detector* (small model with high recall) first identifies potential kicking events based on acceleration and gyroscope data. A *Kick Classifier* (larger model tuned for precision) then confirms these events and assigns a confidence score along with an estimated ball speed.
• **Ball speed estimation:** If a kick is classified, the *Ball Speed Estimator* is triggered to refine the ball speed estimate (km/h) based on the sensor data.
• **Player motion estimation:** The *Player Motion Estimator* continuously processes IMU-derived metrics, estimating speed and distance covered.

#### 2.1.3 Performance metric calculations

• **Absolute distance:** Computed by integrating the estimated speed over time from the Player Motion Estimator for a 5 s interval of the respective training session.
• **Peak speed:** Defined as the maximum speed reached within a 5 s interval of the respective training session, derived from the Player Motion Estimator.
• **Ball speed:** Directly obtained from the Ball Speed Estimator after kick classification.

For the analysis we use the 95th percentile of ball speed, peak speed and distance covered during a training session. We decided not to use the maximum value as it would be prone to outliers. In Section 4.2 we also analyse the results for the 99th percentile to check the dependence of the results on the percentile used.

#### 2.1.4 Subjective metrics: intensity and happiness

To evaluate players’ perceived intensity and happiness after training sessions, a 5-point Likert scale was used (Table 1). This format is widely used in sports and exercise psychology due to its simplicity, ease of interpretation, and demonstrated psychometric robustness. For instance, the Perceived Performance in Team Sports Questionnaire (PPTSQ) also employs a 5-point Likert scale to evaluate athletes’ perceptions of team performance (17).

**Table 1:** Likert scale used for assessing perceived intensity and happiness levels after training sessions.

Likert scale	1	2	3	4	5
Intensity	Very light: effort felt minimal and relaxed	Light: noticeable but easy intensity	Moderate: balanced with highs and lows; challenging	High: demanding; requires recovery time	Very hard: exhausting; recovery is a must
Happiness	Disaster: a day full of mistakes; not my best	Unhappy: below my usual performance	It’s Ok: average performance today	Good: happy with my play today	Perfect: truly proud of my performance

Using an app, players were asked to rate:

1. Intensity: How would you rate the intensity of today’s effort? (1 = Very light, 5 = Very hard)
2. Happiness: How do you feel about your performance? (1 = Disaster, 5 = Perfect)

The intensity scale ranges from *Very light* (minimal effort, relaxed) to *Very hard* (exhausting, requiring recovery). The happiness scale captures emotional responses, from *Disaster* (a frustrating, poor performance) to *Perfect* (an outstanding session). This subjective assessment provides insight into individual player experiences and facilitates correlation analyses with objective performance metrics. Players were instructed to complete the questionnaire independently and immediately after each session. Responses were submitted digitally and individually, without observation or feedback from coaches or teammates. They were informed that their data would remain confidential and be used solely for research purposes, to encourage honest and unbiased reporting. The variable “happiness” was included as a single-item, post-session measure to capture athletes’ affective evaluation of their own performance. Although not based on a standardized psychological scale, the term was explicitly defined in the questionnaire and anchored to a 5-point Likert scale to ensure clarity and interpretability.

### 2.2 Data analysis

#### 2.2.1 Modified Mann-Kendall-test

To detect a monotonic trend in time series data, we decided to use the Mann-Kendall-Test (18, 19). This non-parametric statistical test is widely applied in hydrometeorological studies to analyze trends in variables such as streamflow, water quality, temperature, and precipitation (20). The null Hypothesis ($H_{0}$) assumes that the data is independent and randomly distributed, while the alternative Hypothesis ($H_{1}$) posits the existence of a monotonic trend (increasing or decreasing) in the data.

For a time series with n observations, the test statistic S is calculated as:

$$S=\sum_{i=1}^{n-1}\sum_{\;j=i+1}^{n}\;\text{sign}(x_{j}-x_{i})$$

where

$$\text{sign}(x_{j}-x_{i})=\left\{ \begin{array}{cc} +1,\; & \text{if}\;x_{j}-x_{i}>0,\;\\ 0,\; & \text{if}\;x_{j}-x_{i}=0,\;\\ -1,\; & \text{if}\;x_{j}-x_{i}<0,\; \end{array}\right.$$

and $x_{j}$ and $x_{i}$ are values at time j and i, respectively.

According to (18, 19), the test statistic S approximates a normal distribution when the sample size n≥8. The variance of S under $H_{0}$ is defined as:

$$\text{Var}(S)=\frac{n(n-1)(2n+5)-\sum_{i=1}^{m}T_{i}\cdot i(i-1)(2i+5)}{18},$$

where $T_{i}$ represents the number of tied (equal) values in the i-th group of ties, and m is the total number of groups of tied values. The standardised test statistic Z is computed as:

$$Z=\left\{ \begin{array}{cc} \frac{S-1}{\sqrt{\text{Var}(S)}},\; & \text{if}\;S>0,\;\\ 0,\; & \text{if}\;S=0,\;\\ \frac{S+1}{\sqrt{\text{Var}(S)}},\; & \text{if}\;S<0.\; \end{array}\right.$$

To asses significance, the computed Z value is compared against the critical value from the standard normal distribution for a given significance level α. If $|Z|>Z_{\alpha /2}$, $H_{0}$ is rejected, indicating a statistical significant trend. The direction of the trend is determined by the sign of S: if S>0, the trend is increasing, if S<0 the trend is decreasing.

Because some of the data in our study exhibit autocorrelation, we apply the modified Mann-Kendall test introduced by (21). This modification adjusts the variance of S to account for serial correlation, which can cause the standard Mann-Kendall test to overestimate the significance of trends. While the test statistic S remains unchanged, the variance is modified as follows:

$$\text{Var}(S{)}^{*}=\text{Var}(S)\cdot \frac{n^{*}}{n},$$

where $n^{*}$ is the effective sample size:

$$n^{*}=n\cdot {\left(1+\frac{n(n-1)(n-2)}{2}\sum_{i=1}^{n-1}(n-i)(n-i-1)(n-i-2)\rho_{s}(i)\right)}^{-1}.$$

Here, $\rho_{s}(i)$ denotes the autocorrelation of the ranks at lag i. The adjusted standardised test statistic $Z^{*}$ is then computed as:

$$Z^{*}=\left\{ \begin{array}{cc} \frac{S-1}{\sqrt{\text{Var}(S{)}^{*}}}\; & \text{if}\;S>0\;\\ 0\; & \text{if}\;S=0\;\\ \frac{S+1}{\sqrt{\text{Var}(S{)}^{*}}}\; & \text{if}\;S<0\; \end{array}\right.$$

This adjustment accounts for serial correlation, providing a more accurate assessment of trends in autocorrelated time series and reducing the likelihood of false positives.

#### 2.2.2 Spearmann correlation index

To assess the monotonic relationships between variables, we employed the Spearman correlation coefficient ρ (22):

$$\rho =1-\frac{6\sum_{i=1}^{n}d_{i}^{2}}{n(n^{2}-1)},$$

where n is the number of paired observations and $d_{i}$ is the difference between the ranks of each pair of observations. The Spearman correlation is a non-parametric measure that evaluates the strength and direction of association between two ranked variables. Unlike Pearson’s correlation coefficient, Spearman’s method does not assume a linear relationship or normal distribution of the data, making it particularly suitable for non-linear or non-normally distributed datasets (23). The correlation coefficient ρ ranges from −1 (perfect negative monotonic relationship) to +1 (perfect positive monotonic relationship). A value of 0 indicates no monotonic relationship. We interpret all correlations within the context of their magnitude. Weak but statistically significant correlations are treated as exploratory and interpreted with caution. Given the large number of correlation tests across player positions, teams, subjective variables (intensity, happiness), and performance metrics, we applied the Benjamini-Hochberg procedure (24) to control the false discovery rate (FDR) at α=0.1. This correction was implemented across all Spearman tests per subjective variable. Only correlations with FDR-adjusted p-values below 0.1 were interpreted and visualized.

Although we identified autocorrelation in the time series of some players, we decided against removing trends to calculate the Spearman correlation coefficient. This decision ensures comparability among all players, regardless of the presence of trends in their respective data. Furthermore, some players exhibit gaps in their time series due to illness or injuries, which could complicate or bias detrending methods such as differencing or fitting a trend line. Thus, we opted to retain the original structure of the data for a consistent and unbiased analysis.

### 2.3 Additional analytical considerations

In addition to the specific statistical procedures, several broader analytical decisions were made to ensure robustness and interpretability of the results.

We tested for normality using the Shapiro-Wilk test and found that none of the three performance metrics followed a normal distribution (p-value for all three metrics <0.05). As a result, we confirmed the appropriateness of our non-parametric approach, which included the modified Mann-Kendall trend test and Spearman rank correlation.

Regarding missing data, we decided to not impute missing data, as our focus was on identifying individual-level trends and associations using non-parametric methods. Imputation could introduce artificial stability or variability, especially in longitudinal data with individual-specific trajectories. Furthermore, missingness in our dataset primarily resulted from real-world events such as illness, injury, or absence, which are themselves meaningful in the context of athletic development and training load management. Retaining the original structure of the time series ensured that statistical tests (e.g., modified Mann-Kendall, Spearman correlation) reflected actual measurement patterns rather than imputation artifacts.

While age and experience may influence both subjective assessments and physical performance, these factors are implicitly accounted for by analyzing players separately within the U17 and U20 team groups. We chose not to include additional control variables or compute partial correlations, as our focus was on exploratory, subgroup-level insights and individual variability. This approach also allowed us to maintain comparability across players with varying levels of data completeness due to illness or injury.

All analyses were conducted using the software R (25), employing the *mmkh* function from the modifiedmk package (26) for the modified Mann–Kendall trend test. Spearman correlations and multiple testing corrections using the Benjamini–Hochberg procedure (24) were performed with functions from the base stats package, specifically *cor.test* and *p.adjust*. Visualizations were created using the ggplot2 package (27).

## 3 Results

### 3.1 Descriptive analysis

Table 2 presents the descriptive statistics for the key performance metrics—peak speed, absolute distance, and ball speed—across two teams (U17 and U20) over the analyzed period (November 7, 2023, to January 10, 2025). The statistics include the mean, standard deviation (sd), median, interquartile range (IQR), minimum (min), and maximum (max) values.

**Table 2:** Descriptive statistics of performance metrics; time range: 2023/11/07 to 2025/01/10, IQR = Q3−Q1; ball speed is measured as an integer; peak speed and absolute distance are motion metrics and represent the respective value in a 5 s interval; sd stands for standard deviation; IQR stands for interquartile range; min stands for the minimum and max for the maximum measured value. The maximum value of the peak speed comes from the same player, as she has trained in both teams (U17 and U20).

Variable	Team	Mean	Sd	Median	IQR	Min	Max
Peak speed (km/h)	U17	7.96	3.53	6.99	4.77	3.61	32.51
Peak speed (km/h)	U20	7.95	3.63	6.82	4.87	3.60	32.51
Absolute distance (m)	U17	8.49	3.35	7.39	3.87	5.00	38.75
Absolute distance (m)	U20	8.51	3.42	7.37	3.74	5.00	41.25
Ball speed (km/h)	U17	51.51	11.97	49.00	13.00	30.00	115.00
Ball speed (km/h)	U20	52.34	12.30	50.00	14.00	30.00	110.00

Across both teams, the mean peak speed values reached in 5 s intervals are comparable (U17: 7.96 km/h; U20: 7.95 km/h), with similar dispersion as indicated by the standard deviations (3.53 and 3.63, respectively). The absolute distance covered in a 5 s interval shows slightly higher mean values in U20 (8.51 m) compared to U17 (8.49 m), although the median values remain similar (7.39 m vs. 7.37 m). The IQR for absolute distance suggests that the distribution of this variable is relatively consistent across both teams, with moderate variability.

Ball speed shows higher overall variability compared to the other metrics, as indicated by its larger standard deviations and IQRs. The mean ball speed is slightly higher in U20 (52.34 km/h) than in U17 (51.51 km/h), with a broader range of values in both teams, spanning from 30 km/h (below 30 km/h, the data is not taken into account as the movement is not reliable enough at extremely low speeds) to over 110 km/h.

These descriptive statistics provide a fundamental understanding of the distribution and variability of performance metrics within each team, laying the foundation for further correlation and trend analyses.

**Figure 4:**
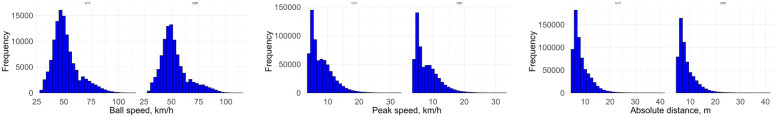
Histogramms of performance metrics per team, time range: 2023/11/07 to 2025/01/10.

**Figure 5:**
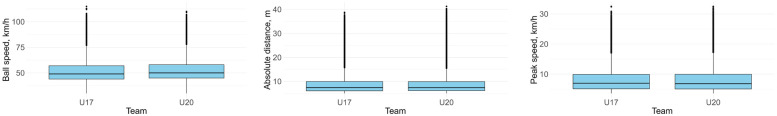
Boxplots of performance metrics per team, time range: 2023/11/07 to 2025/01/10; ball speed is measured as an integer; peak speed and absolute distance are motion metrics and represent the respective value in a 5 s interval.

Figures 4, 5 complement the descriptive statistics presented in Table 2 by visually illustrating the distribution and variability of the three key performance metrics (peak speed, absolute distance, and ball speed) across the U17 and U20 teams during the analyzed period (November 7, 2023, to January 10, 2025).

The histograms in Figure 4 provide a detailed view of the frequency distributions for each metric. For ball speed, both teams exhibit a unimodal distribution with a concentration around 50–60 km/h, though U20 displays slightly higher frequencies at higher speeds compared to U17. Peak speed follows a right-skewed distribution for both teams, with most values clustered below 10 km/h. Similarly, absolute distance shows a pronounced right-skewed pattern, with most observations below 15 m for both teams. These histograms highlight the overall trends and differences in performance metrics between the two age groups.

The boxplots in Figure 5 further emphasize the variability and central tendencies of these metrics. For ball speed, U20 demonstrates slightly higher median values and a broader range compared to U17, consistent with the descriptive statistics. Peak speed boxplots reveal similar medians for both teams but show slightly more outliers in U20, indicating occasional higher peak speeds. Absolute distance boxplots confirm comparable medians across teams but also illustrate greater variability in U20 through a larger spread and more outliers.

These visualizations corroborate the statistical findings and provide deeper insights into the distributional characteristics of performance metrics across age groups, enabling a comprehensive understanding of team-specific trends in soccer performance.

### 3.2 Longitudinal trend analysis

**Figure 6:**
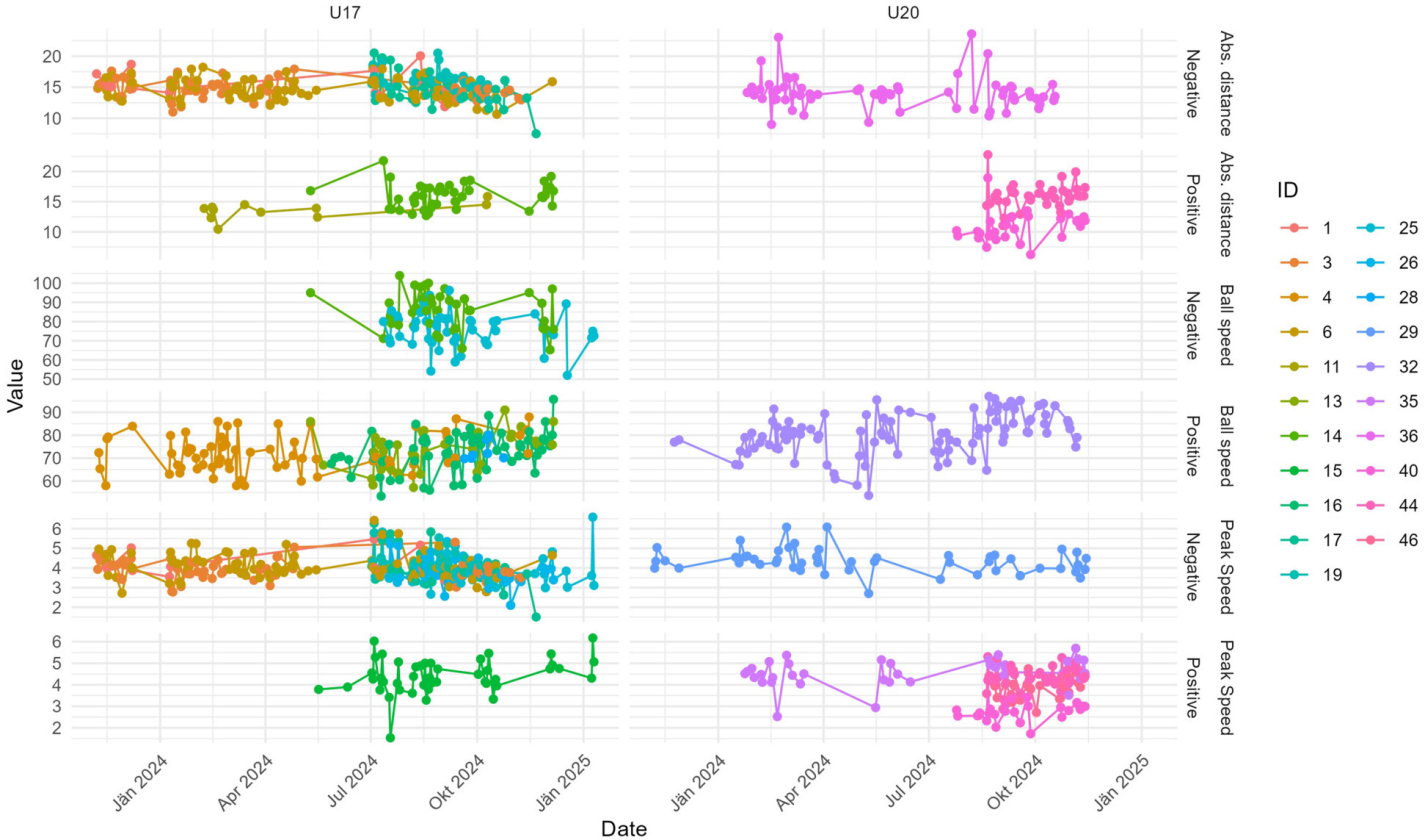
Statistical significant trends per player per the 95th percentile of the performance metric; time range: 2023/11/07 to 2025/01/10; statistical test: modified Mann-Kendall test p<0.05.

Figure 6 shows statistically significant trends in the 95th percentile of the performance metrics for individual players over the period from November 7, 2023, to January 10, 2025. The modified Mann-Kendall test (p<0.05) was used to identify these trends. The data is grouped into two sets, labeled U17 and U20, with each subplot representing a specific performance metric, further divided into positive and negative trends. Each player’s data is color-coded by their ID for clarity.

Table 3 provides a summary of the results in Figure 6 and shows the significant trends across the three performance metrics: ball speed, peak speed, and absolute distance. For 21 out of 46 players, a total of 30 significant trends were identified, with 16 negative and 14 positive trends. The distribution reveals that peak speed has the highest number of significant trends (13), with a majority being negative (8). Ball speed exhibits the fewest trends (7), dominated by positive changes (5), while absolute distance shows more negative trends (6) than positive (4). In addition to trend direction and significance, we report Kendall’s tau and Sen’s slope estimates for players with significant trends to quantify trend strength and magnitude in Table 4.

**Table 3:** Statistical significant trends per the 95th percentile of the performance metric summarised for performance metrics; time range: 2023/11/07 to 2025/01/10; statistical test: modified Mann-Kendall test *p* < 0.05.

Performance metric	Negative	Positive	Total
Ball speed	2	5	7
Peak speed	8	5	13
Absolute distance	6	4	10
Total	16	14	30

**Table 4:** Summary of statistically significant trends in performance metrics per player based on the modified Mann-Kendall test. Reported values include the corrected p-value (after variance correction), Sen’s slope (indicating magnitude of trend per time unit), and Kendall’s tau (measuring monotonic trend strength). Direction indicates whether the trend was positive (increasing) or negative (decreasing).

ID	Performance metric	Corrected p-value	Sen’s slope	Tau	Direction
4	Ball speed	0.023	0.087	0.166	Positive
13	Ball speed	0.004	0.279	0.288	Positive
14	Ball speed	0.049	−0.187	−0.195	Negative
16	Ball speed	0.006	0.111	0.230	Positive
25	Ball speed	0.000	−0.114	−0.175	Negative
28	Ball speed	0.017	0.281	0.484	Positive
32	Ball speed	0.000	0.101	0.289	Positive
1	Peak speed	0.003	−0.027	−0.233	Negative
3	Peak speed	0.035	−0.005	−0.156	Negative
6	Peak speed	0.005	−0.006	−0.189	Negative
15	Peak speed	0.045	0.012	0.190	Positive
17	Peak speed	0.000	−0.010	−0.321	Negative
19	Peak speed	0.020	−0.008	−0.239	Negative
25	Peak speed	0.045	−0.006	−0.129	Negative
26	Peak speed	0.004	−0.010	−0.222	Negative
29	Peak speed	0.020	−0.010	−0.214	Negative
35	Peak speed	0.045	0.010	0.201	Positive
40	Peak speed	0.009	0.010	0.232	Positive
44	Peak speed	0.000	0.004	0.106	Positive
46	Peak speed	0.030	0.012	0.182	Positive
1	Abs. distance	0.000	−0.127	−0.250	Negative
3	Abs. distance	0.001	−0.024	−0.233	Negative
6	Abs. distance	0.006	−0.023	−0.225	Negative
11	Abs. distance	0.023	0.190	0.487	Positive
14	Abs. distance	0.000	0.042	0.195	Positive
17	Abs. distance	0.000	−0.045	−0.304	Negative
19	Abs. distance	0.011	−0.051	−0.315	Negative
36	Abs. distance	0.033	−0.015	−0.169	Negative
40	Abs. distance	0.035	0.063	0.240	Positive
44	Abs. distance	0.013	0.026	0.190	Positive

### 3.3 Correlation analysis

**Figure 7:**
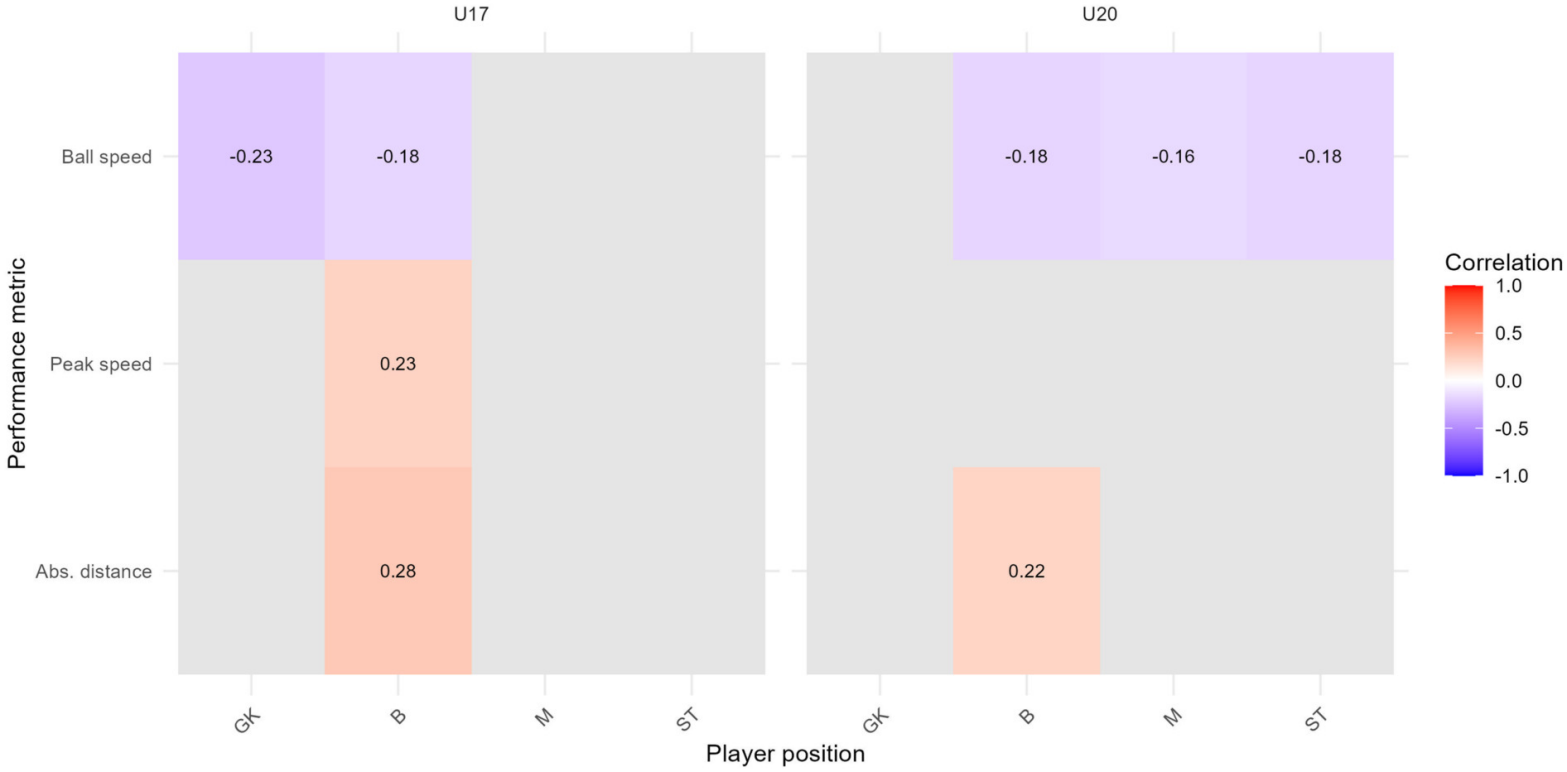
FDR-corrected correlation heatmap between subjective assessment of intensity and the 95th percentile of performance metrics, stratified by team and player position; time range: 2023/11/07 to 2025/01/10; Spearman correlation index with FDR correction (p<0.1).

**Figure 8:**
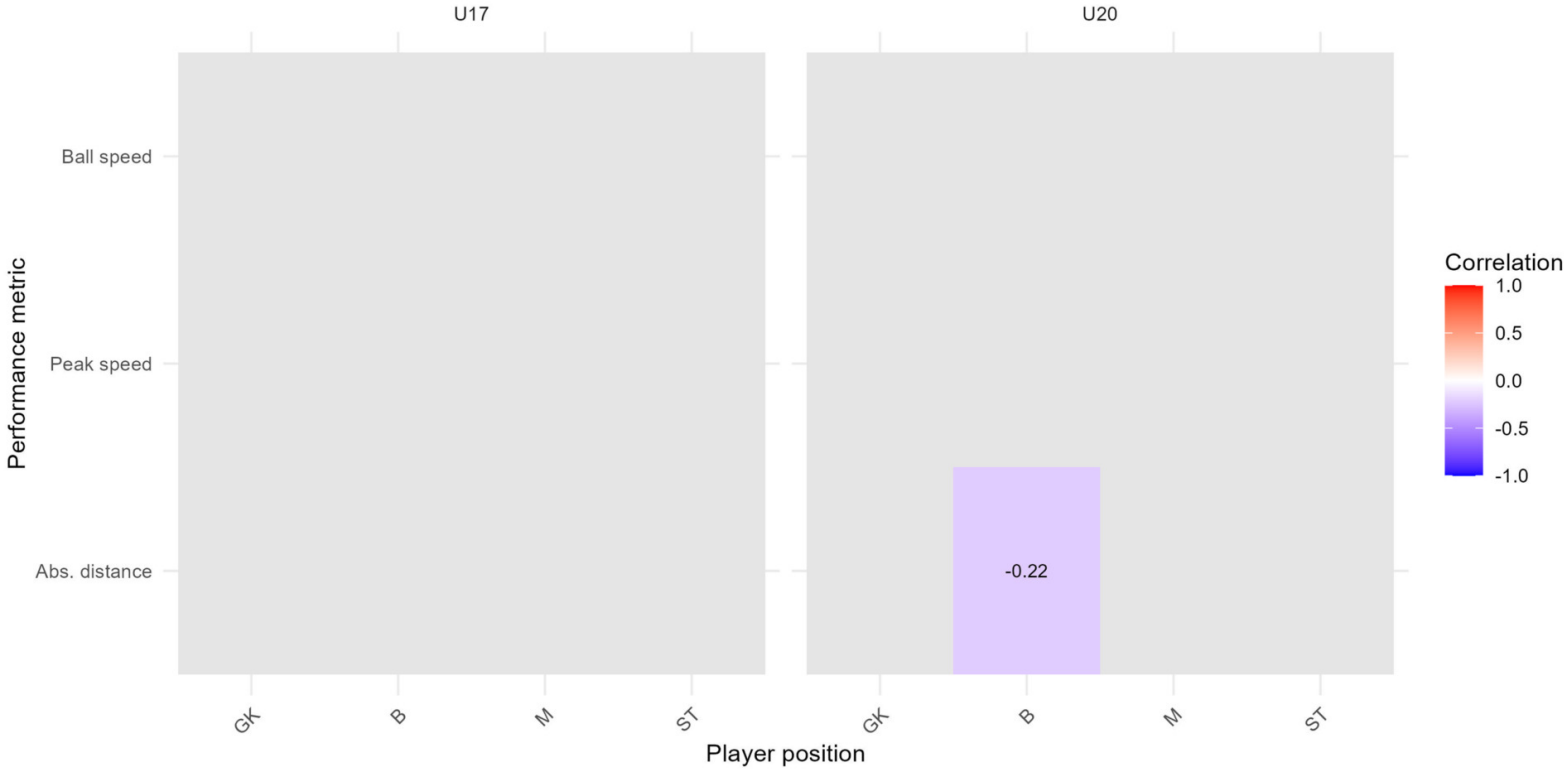
FDR-corrected correlation heatmap between subjective assessment of happiness and the 95th percentile of performance metrics, stratified by team and player position; time range: 2023/11/07 to 2025/01/10; Spearman correlation index with FDR correction (p<0.1).

Figures 7, 8 show correlation heatmaps based on FDR-adjusted p-values, illustrating the relationships between subjective assessments (intensity and happiness) and the 95th percentile of performance metrics (ball speed, absolute distance, and peak speed) for players grouped by team and position. The heatmaps are divided into two panels representing the two teams (U17 and U20), with player positions on the *x*-axis and performance metrics on the *y*-axis. Positive correlations are shown in red hues, negative correlations in blue hues, and only those combinations with FDR-adjusted p-values below 0.1 are displayed.

Figure 7 shows statistically significant (p<0.1) negative correlations between perceived intensity and ball speed for goalkeepers (U17, ρ=−0.23) and defenders (U17, ρ=−0.18; U20, ρ=−0.18). Positive correlations were found between intensity and peak speed (U17, defenders, ρ=0.23) and absolute distance (U17, defenders, ρ=0.28; U20, defenders, ρ=0.22). No other combinations met the corrected significance threshold.

In Figure 8, only one statistically significant correlation remains after FDR correction: a negative association between happiness and absolute distance for defenders in the U20 team (ρ=−0.22). All other correlations fell above the FDR-adjusted threshold.

## 4 Discussion

### 4.1 Main results

Our findings provide valuable insights into the longitudinal trends and correlations of performance metrics in professional soccer players, revealing key patterns and potential implications for training and performance evaluation.

The descriptive statistics and visualizations presented in Table 2 and Figures 4, 5 provide valuable insights into the performance metrics of U17 and U20 soccer teams over the analyzed period. These findings highlight both similarities and differences in physical performance characteristics between the two age groups.

The comparable mean peak speed values across U17 and U20 teams (7.96 m/s vs. 7.95 m/s) suggest that this metric may not significantly differentiate between age groups at these levels. However, the slightly higher number of outliers observed in U20 (Figure 5) indicates that older players occasionally achieve higher peak speeds, which could reflect differences in individual physical development.

The slightly higher mean absolute distance covered by U20 players (8.51 m vs. 8.49 m) aligns with expectations that older players may engage in more extensive movement patterns due to increased training intensity. The greater variability observed in U20 (Figure 5) further supports this hypothesis, suggesting that individual differences in endurance or playing style may become more pronounced with age.

The higher mean ball speed observed in U20 (52.34 km/h vs. 51.51 km/h) and its broader range (Figure 4) indicate that older players may possess superior technical skills or physical strength, enabling them to execute faster passes or shots. This finding is consistent with the notion that ball speed is a critical performance metric influenced by both technical proficiency and physical maturity.

The longitudinal trend analysis can be interpreted as modeling individual response trajectories over time, capturing athlete-specific performance development based on longitudinal insole-derived data. This approach aligns with current recommendations in athlete monitoring that emphasize intra-individual variation and personalized interpretation. The analysis revealed distinct patterns in player performance metrics, with peak speed showing the highest number of significant trends, predominantly negative. This decline could indicate cumulative fatigue over the study period, possibly linked to intensified match schedules or insufficient recovery time. Conversely, the positive trends observed in ball speed suggest targeted improvements in skill-specific training. The distribution of trends across metrics and teams underscores the need for tailored interventions, as team-specific factors, such as coaching strategies or player composition, likely influence outcomes.

As shown in Figure 7, the FDR-corrected correlation analysis revealed selective but interpretable relationships between subjective intensity assessments and performance metrics. Only a limited number of associations reached statistical significance after correction, but the observed patterns offer exploratory insights into how perceived exertion relates to objective performance in specific roles and teams. For example, significant negative correlations between intensity and ball speed were evident for all player positions, suggesting that higher perceived effort may relate to reduced ball handling efficiency, possibly due to fatigue or training context. A positive association was found between intensity and absolute distance among U17 and U20 defenders, indicating that higher self-reported intensity can reflect increased physical output in certain defensive roles.

Figure 8 shows FDR-corrected associations between post-session happiness and performance metrics. In the U20 team, defenders show significant negative correlations between happiness and absolute distance. Additional a negative correlation were found for defenders and midfielders when using the 99th percentile of peak speed and absolute distance. These findings suggest that elevated physical output may not always correspond to greater subjective satisfaction, especially in roles that emphasize tactical stability over physical explosiveness. Conversely, U17 midfielders showed a positive correlation between happiness and ball speed, suggesting that technical performance may influence perceived satisfaction in more offensively oriented roles. These position- and age-specific results highlight the nuanced relationship between affective responses and physical output in high-performance settings.

For practitioners, these findings offer practical implications for individualized training design. The descriptive differences between U17 and U20 players support the implementation of age-specific interventions to improve peak speed, endurance, and technical execution. Tactical and role-based conditioning may be especially relevant for defenders and goalkeepers, while offensively positioned players could benefit from dynamic, performance-driven drills.

The FDR-corrected results also emphasize the psychological dimension of training. Defenders who experience lower satisfaction following physically intense sessions may benefit from targeted exercises that promote engagement, communication, and perceived relevance. In contrast, players whose satisfaction is positively associated with performance outputs, such as U17 defenders, may respond best to technically focused, feedback-rich environments.

In summary, although few correlations met statistical significance under FDR correction, the observed patterns offer robust, role-specific insight into the interplay between subjective perception and physical performance. These findings reinforce the value of integrating wearable-derived metrics with psychological self-assessments to optimize training effectiveness and athlete development. However, we emphasize that these findings are exploratory and should be interpreted as preliminary indications of potential associations, which warrant further investigation in future research.

### 4.2 Robustness check

**Figure 9:**
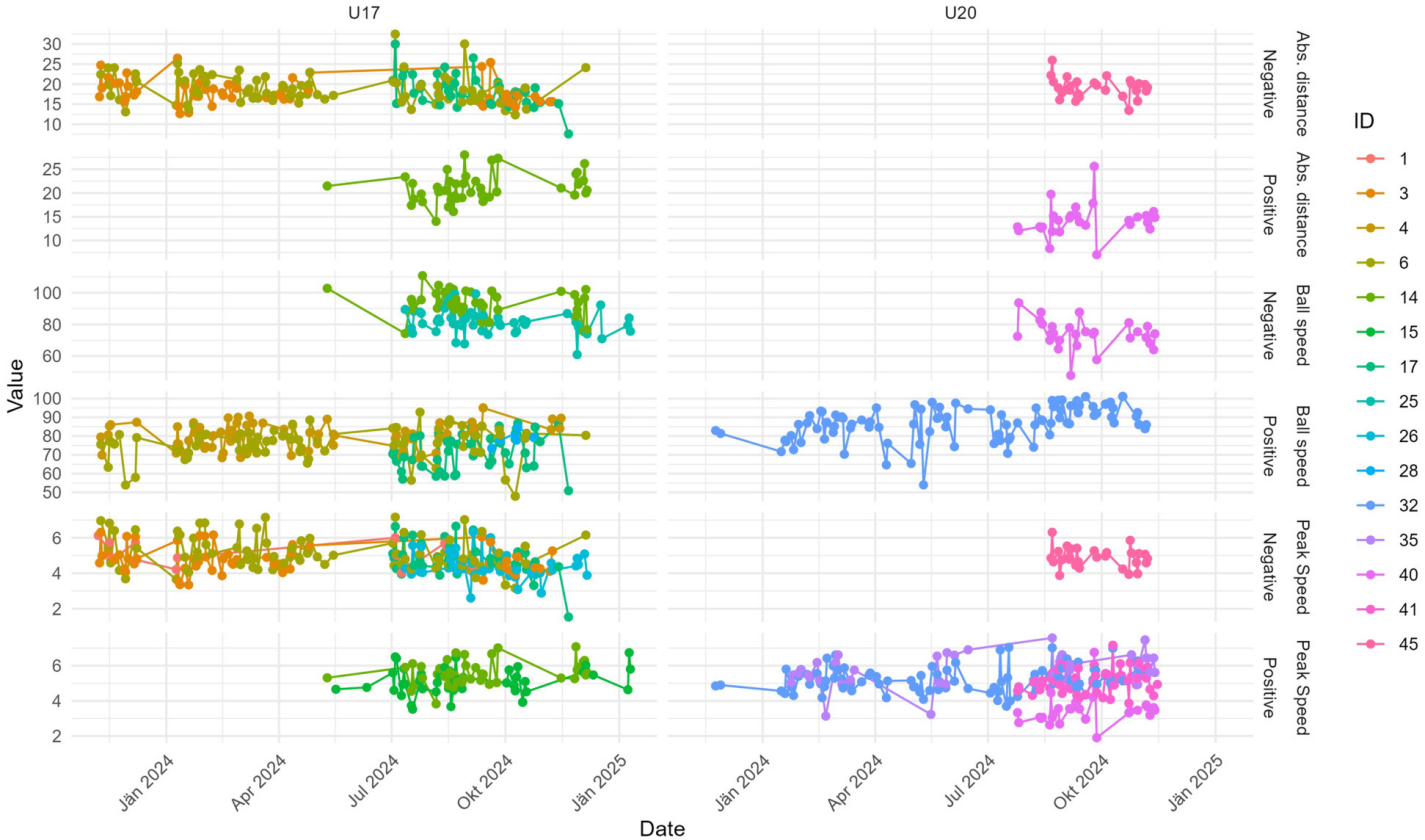
Statistical significant trends per player per the 99th percentile of the performance metric; time range: 2023/11/07 to 2025/01/10; statistical test: modified Mann-Kendall test p<0.05.

To assess the robustness of the observed statistical relationships, we first repeated the modified Mann-Kendall trend analysis using the 99th percentile of each performance metric. The results, summarized in Table 5 and visualized in Figure 9, show a total of 26 statistically significant trends, slightly fewer than the 30 identified at the 95th percentile (Table 3). Peak speed continues to exhibit the highest number of significant trends (12), though the distribution is now evenly split between positive and negative trends (6 each), suggesting more variability in extreme speed outputs over time. Ball speed trends remain predominantly positive (5 vs. 3 negative), while absolute distance shows a slight skew toward negative trends (4 vs. 2 positive), indicating a possible decline in maximal distance efforts across the period.

**Table 5:** Statistical significant trends per the 99th percentile of the performance metric summarised for performance metrics; time range: 2023/11/07 to 2025/01/10; statistical test: modified Mann-Kendall test *p* < 0.05.

Performance metric	Negative	Positive	Total
Ball speed	3	5	8
Peak speed	6	6	12
Absolute distance	4	2	6
Total	13	13	26

The spatial and temporal distribution of these trends across both U17 and U20 teams remains consistent with the original analysis, reinforcing the validity of the observed individual development patterns. The replication of trend directionality, especially the dominance of peak speed trends, further supports the robustness of the original findings and confirms that the 95th percentile results are not overly sensitive to the specific percentile threshold used.

**Figure 10:**
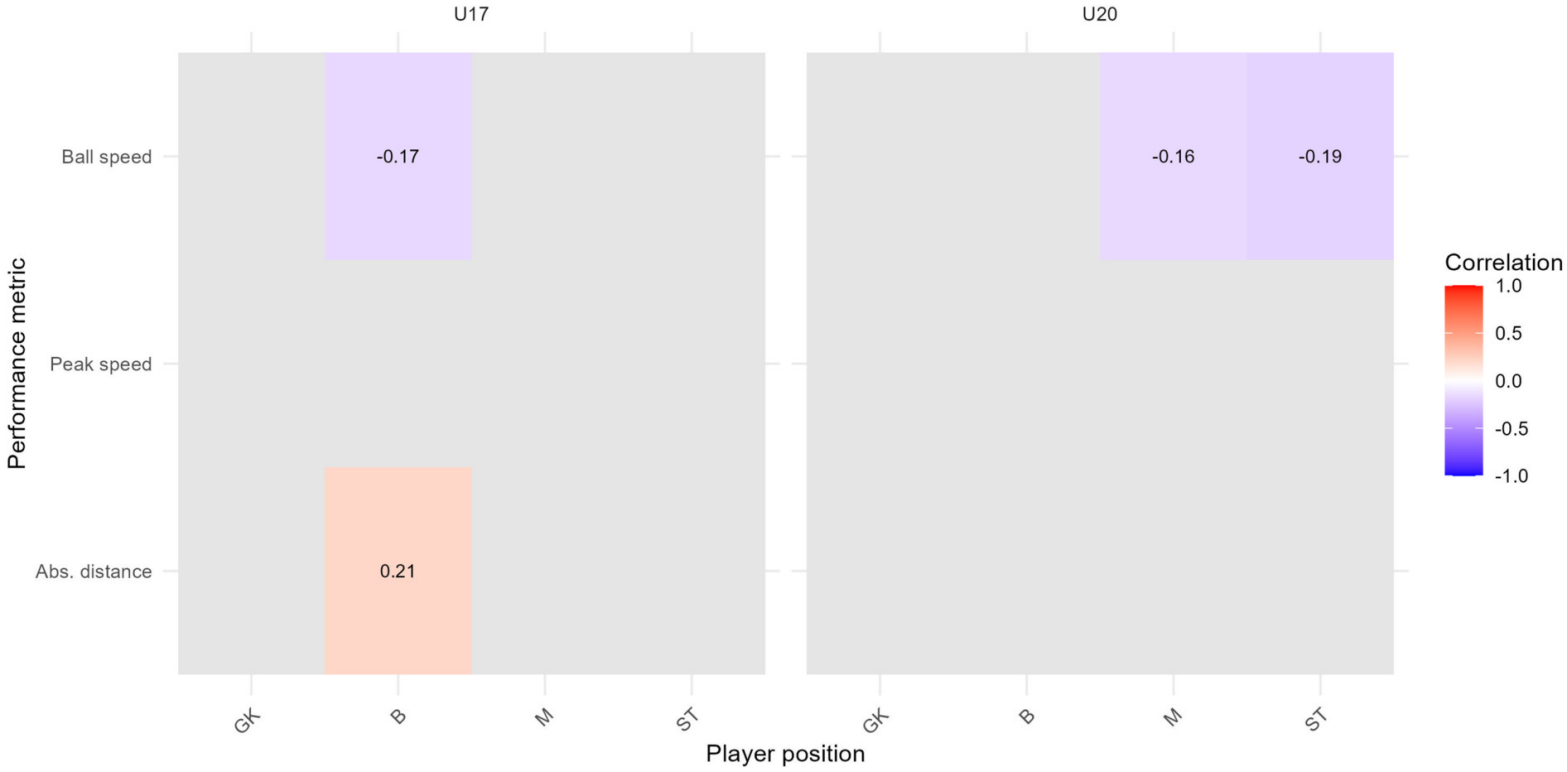
FDR-corrected correlation heat map for the subjective assessment of intensity and the 99th percentile of performance metrics per player position and team; time range: 2023/11/07 to 2025/01/10; Spearman correlation index with FDR correction (p<0.1); GK, goalkeepers; B, defenders; M, midfielders; ST, strikers.

**Figure 11:**
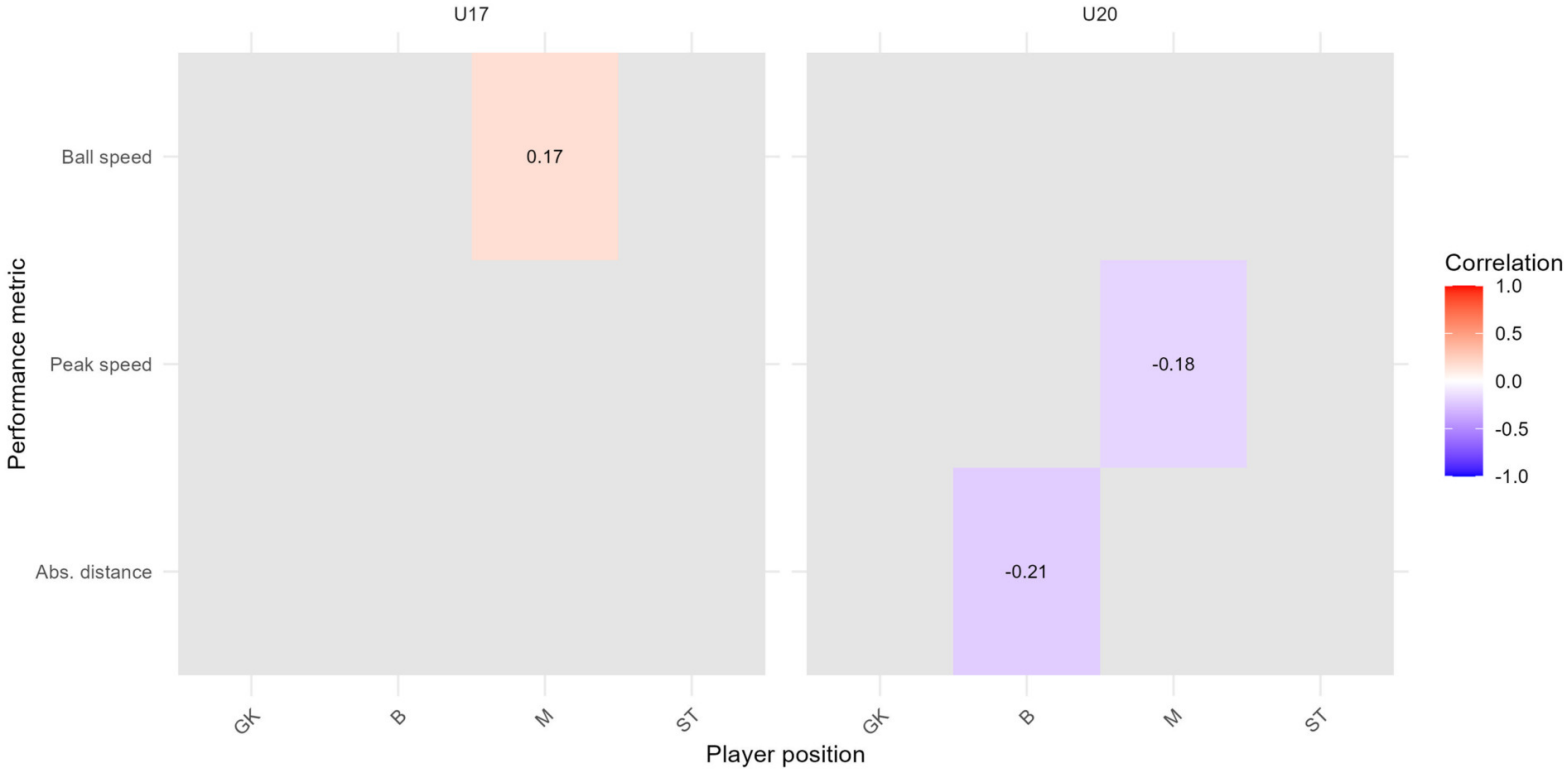
FDR-corrected correlation heat map for the subjective assessment of happiness and the 99th percentile of performance metrics per player position and team; time range: 2023/11/07 to 2025/01/10; Spearman correlation index with FDR correction (p<0.1); GK, goalkeepers; B, defenders; M, midfielders; ST, strikers.

In addition, we replicated the Spearman correlation analysis (applying FDR correction) from Section 3.3 using the 99th percentile of each performance metric, to examine whether the associations between subjective assessments (intensity and happiness) and physical performance remain consistent under more extreme conditions. As illustrated in Figures 10, 11, the overall pattern of statistically significant relationships becomes more selective at the 99th percentile level.

For subjective intensity (Figure 10), negative correlations with ball speed are still evident for defenders (U17, ρ=−0.17) midfielders (U20, ρ=−0.16) and strikers (U20, ρ=−0.19). A statistically significant positive correlation is observed between intensity and absolute distance for defenders in the U17 team (ρ=0.21). No other player positions or performance metrics met the corrected significance threshold.

For subjective happiness (Figure 11), more distinct effects emerged. Notably, defenders in the U20 team show negative correlations with absolute distance (ρ=−0.21). Additionally, midfielders in the U20 team show negative correlations with peak speed (ρ=−0.18), while midfielders in the U17 show a positive correlation with ball speed (ρ=0.17).

Overall, the consistency of both longitudinal trend results and selected correlation structures at the 99th percentile underscores the robustness of our analytical framework. While fewer associations reached statistical significance, key relationships, such as the negative link between intensity and ball speed or the negative association between happiness and performance in U20 players, persisted. These findings indicate that the relationships between subjective perceptions and objective performance metrics are not artifacts of percentile selection but rather reflect role- and context-dependent mechanisms that become particularly evident under extreme performance conditions.

The use of the 95th and 99th percentiles in performance analysis represents a methodological trade-off between capturing high-performance output and maintaining robustness against variability. While the 95th percentile provides a stable estimate of upper performance under typical conditions, the 99th percentile emphasizes near-maximal efforts. In our study, the 99th percentile was used as a percentile-based summary statistic and not as a raw maximum value, what reduces the influence of individual outliers. Combined with FDR correction, this approach mitigates the risk of spurious associations. Nonetheless, the reduced number of statistically significant correlations at the 99th percentile highlights the inherent tension: higher specificity and sensitivity to peak performance may come at the cost of reduced statistical power and generalizability. As such, percentile selection should be guided by the intended analytical objective, whether to monitor consistent high-level output or to isolate peak expressions of individual capacity.

As an additional robustness check, we repeated the Spearman correlation analysis using only players who were uniquely assigned to either the U17 or U20 team, thereby excluding two players who participated in both datasets. One of these players was categorized as a defender or midfielder, and the other as a striker. The resulting correlation heatmap for subjective intensity is provided as Supplementary Figure S1. The main correlation patterns remained largely consistent in both direction and magnitude. Notably, the significant negative associations between intensity and ball speed persisted, as did the positive associations between intensity and absolute distance and peak speed. Interestingly, the previously significant correlation between intensity and ball speed for defenders disappeared, while two new significant correlations emerged for U17 midfielders (with peak speed and absolute distance), and one new correlation appeared for U20 strikers (with absolute distance).

No statistically significant correlations were observed for subjective happiness in this reduced dataset. Overall, these results support the robustness of the main findings and suggest that the inclusion of overlapping players did not introduce bias into the observed correlation structures.

### 4.3 Limitations and outlook

This study is limited by its focus on a specific timeframe and the focus on two teams, which restricts the generalisability of the findings. Training culture, coaching style, and organizational context may vary substantially across clubs or national systems. Consequently, the observed patterns should be seen as exploratory and context-dependent. Future research should aim to replicate this approach across broader samples, including different clubs and regions, competitive levels and training environments.

In addition, the study is limited by its reliance on the 95th percentile of performance metrics, potentially missing trends in other data percentiles. So we added an analysis where we analyse the results for the 99th percentile of performance metrics. Subjective intensity and happiness assessments may be influenced by psychological factors, introducing bias. Statistical methods such as the modified Mann-Kendall test and Spearman correlation, while appropriate, could be complemented by machine learning to uncover nonlinear patterns. Future research should explore longer timeframes, integrate objective and psychological metrics, assess external factors like weather or match difficulty, include injury data, and expand the sample to teams from diverse leagues or regions for broader insights.

Although our study focused on female youth soccer players, the underlying principles and methodology have potential relevance in other performance contexts. The combination of wearable-based motion tracking and post-session subjective assessment could be similarly applied in male football or other team sports such as basketball or handball, where movement patterns, psychological engagement, and role-specific demands are also critical. Moreover, while our analysis focuses exclusively on training sessions, it is important to note that subjective perceptions such as intensity or happiness may still be influenced by role-based and contextual factors. For example, players who regularly start in matches may approach training with different goals, expectations, or mental loads compared to those who are primarily substitutes. Moreover, variations in training focus (e.g., recovery vs. conditioning), individual readiness, or anticipation of selection could shape how players perceive and report exertion or affect. Although we did not systematically collect role-related metadata, these factors may contribute to within-group variation in subjective assessments and should be considered in future research.

A further limitation lies in the accuracy of the machine learning models embedded in the TEAM-FX system. Although partially validated in a previous studies (15, 16), these models may still produce classification errors such as false positives or false negatives in kick detection, as well as minor estimation inaccuracies in derived metrics like ball speed. Such errors could introduce noise at the event level, especially in shorter time windows, and may attenuate the strength of correlations with subjective assessments. While we relied on validated output formats and summary statistics to mitigate this, these model-based uncertainties should be kept in mind when interpreting the results.

In addition, future research may benefit from applying longitudinal modeling techniques such as latent growth models. These models allow for the estimation of both fixed and random effects across time and could provide a more nuanced understanding of intra- and inter-individual variability in subjective and objective performance measures, particularly when data with regular time intervals and higher completeness become available.

Moreover, although we used non-parametric methods to capture individual-level variation without distributional assumptions, future studies may consider applying generalized linear mixed-effects models to more formally account for hierarchical data structures and repeated measures. This could offer complementary insights, especially when larger datasets become available.

In conclusion, this study underscores the importance of monitoring performance trends and their relationship with perceived intensity. By integrating these insights into training and match preparation, teams can better optimize player performance and reduce injury risks over the long term.

## 5 Conclusion

This study aimed to identify and understand trends in key performance metrics and their correlations with subjective intensity and happiness assessments among players in two football teams over a 14-month period. The detection of significant monotonic trends across multiple performance metrics highlights the potential of longitudinal monitoring to inform individual development. The observed correlations between subjective intensity and happiness ratings and objective metrics, as well as the positional differences in these relationships, suggest initial, context-sensitive connections between perceived effort and performance outcomes. The identified weak correlations offer exploratory insights into the interplay between perceived and measured performance. Building on these findings, future studies may apply this integrated approach to other sports disciplines, age groups, and competition levels to explore its broader applicability. The findings may support sports practice by encouraging the combined use of wearables and subjective self-assessments to help adjust training loads, monitor psychological responses, and guide the individualized development of athletes. This approach may also hold value for elite training programs seeking to individualize performance monitoring and optimize athlete readiness.

## Data Availability

The data analyzed in this study is subject to the following licenses/restrictions: Third party licensing restrictions apply. Requests to access these datasets should be directed to Burkhard Duemler, burkhard.duemler@adidas.com.
