# Supplementary material for: Performance tracking in female youth soccer through wearables and subjective assessments
Source: Front Sports Act Living. 2025 Jun 30;7:1627820. doi: 10.3389/fspor.2025.1627820 (PMC12258290; doi:10.3389/fspor.2025.1627820)
Supplement: Supplementary file 1 [file Datasheet1.pdf]

Supplementary Material

1 SUPPLEMENTARY TABLES AND FIGURES

1.1 Figures

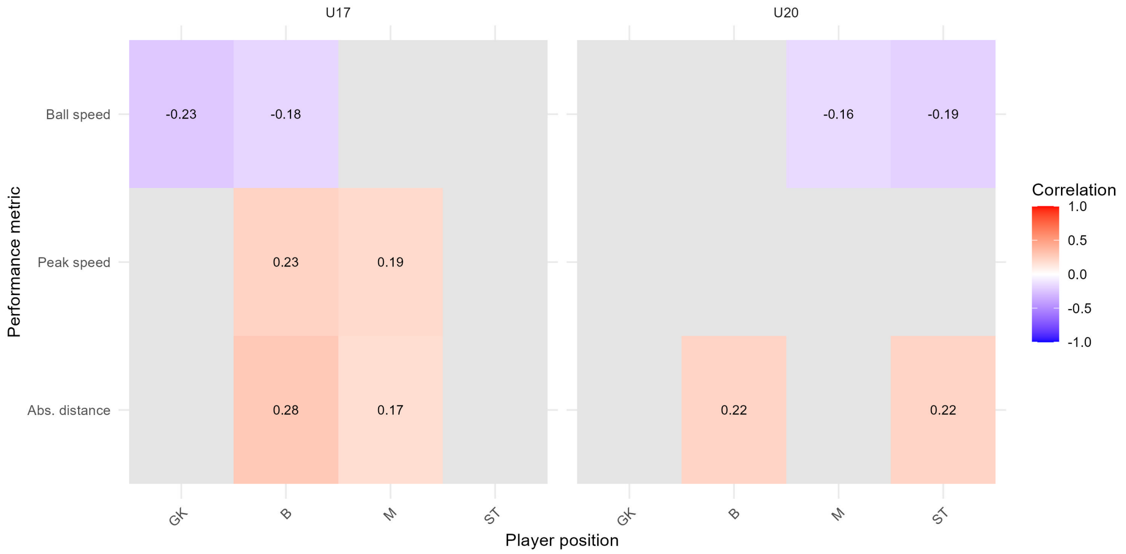

Figure S1. FDR-corrected correlation heatmap for subjective intensity and the 95th percentile of performance metrics after excluding the two players who appeared in both the U17 and U20 datasets; time range: 2023-11-07 to 2025-01-10; Spearman correlation index with FDR correction ($p_{\mathrm{FDR}} < 0.1$); GK: goalkeepers, B: defenders, M: midfielders, ST: strikers.
